# Progress

**Published:** 1914-07

**Authors:** 


PROGRESS.—New conceptions of some old diseases are of great interest and import. Our attitude toward pneumonia has changed most radically in a few years. Now we direct our efforts toward the intestinal tract, recognizing that it is from that source that disaster is perhaps most apt to come. We restrict or eliminate putrefactive food elements. Some men are washing out the colon, though we confess that this seems to us a formidable procedure in pneumonia. The alleged utility of guaiacol carbonate in this disease would seem to depend upon its inhibitory effect upon the growth of the pneumococci in the feces. Experiments are being made in various quarters looking to the inhibition of pneumococcus growth in feces by various agents, with a view to the discovery of the most active. During pneumonia the pneumococcus predominates among the intestinal flora. In a number of series of cases the carrying out of the foregoing principles has given astonishingly low mortalities.

And so with other diseases; pernicious anemia, for example, which bids fair to be classed among the germ affections. Many conditions that used to be called rheumatism are now clearly differentiated. It will not be long before our ideas will be crystallized on the subject of eclampsia, confusing though they are just at present.

Many of the older conceptions are being "knocked into cocked hats." The case of rheumatism is especially interesting. We are familiar with the parts played by the gonococcus and the streptococcus, and Poncet and Stock have shown that tuberculosis is at the bottom of some of the so-called rheumatism, tuberculin tests causing characteristic reactions in the affected joints.—*Medical Times.*

